# Non-Intrusive Load Monitoring

**DOI:** 10.3390/s22176675

**Published:** 2022-09-03

**Authors:** Luigi Fortuna, Arturo Buscarino

**Affiliations:** 1 Dipartimento di Ingegneria Elettrica Elettronica e Informatica, University of Catania, 95125 Catania, Italy; 2 IASI, Consiglio Nazionale delle Ricerche (CNR), 00185 Roma, Italy

Non-Intrusive load monitoring (NILM) represents an emerging strategy based on the application of sevaral multidisciplinary topics. The core of NILM systems is based on the following principle. Let P(t) be the aggregated energy, defined as the summation of *n* terms $p_{i}\left(t\right)$ representing single active users at time *t* in a network of electrical loads. The task is to estimate the single terms, $p_{i}\left(t\right)$, from P(t) without dedicated sensors or, at most, by introducing a few sensors. For a journal such as *Sensors*, it is therefore outstanding that many papers on the topic appeared recently, since the NILM approach tends to save the quantity of sensors, despite introducing systems able to completely monitor and make forecasts based on algorithms for data reconstruction. An NILM system can be, therefore, intended as a soft sensor devoted to the blind separation of energy signals. What is important in the topic is the role of the data handling and that of software architectures, since they must suitably support disaggregating algorithms coupled to forecasting and planning strategies.

The concept of energy planning, the conception of smart sustainable cities based on green technologies, the efforts that must be invested in order to design systems reliable for energy saving and forecasting, thus reducing the electronic equipment and therefore the number of sensors, are all topics faced by both architects and system engineers, and these involve NILM systems. For a comprehensive survey on the topic and on the advanced concepts related to NILM systems applied to energy disaggregation and planning, we refer to the paper by Kaselimi et al. [1].

The hidden common characteristic of all NILM approaches is that they must contain, at least, an almost complete model of the global electrical network. In classic control systems, the idea of observing the complete state of a system with many variables by using only a small number of measured quantities, such as the inputs and the outputs, is often adopted in order in order to have a reliable compensator. Indeed the NILM strategies display some analogies with the concept of the observer.

In the following, the details of specific papers on the NILM problem, recently appeared on *Sensors*, are discussed.

In the contribution by Zhou et al. [2], NILM is conceived in terms of advanced-learning machine systems organized in two levels. The first one (TTRnet) is related to the Input Embedding, structured with more modules, such as transformers, temporal pooling, and the rethinkNet module. The second part is concerned with the so-called Multi-Label Focal Loss (MLFL), which is devoted to improving the weights separately. The efficiency of this complex structure is proved with more experiments produced by using the Commercial Building Dataset (COMBET). The authors present both pre-processing data results and suitable evaluation metrics. The results are discussed also in comparison with a real-world scenario. The discussion about the performance of the study of the presented NILM system is referred to the simultaneous monitoring of multiple commercial loads. Thanks to the performance of the proposed architecture, the perspective of integrating more presented learning machines for the aggregation of more commercial loads is positively received. Another approach to NILM based on artificial neural networks has been proposed in the study by Hur et al. [3] with the aim of improving the robustness of energy disaggregation in the presence of noisy measurements.

The paper by Massida and Marrocu [4] looks at the NILM problem in terms of Bayesian estimation algorithms. The authors explain very clearly the physical problem characterizing electrical-energy consumption. The analytical details of the load calculation are well presented, producing a reliable engineering model. The study is devoted to an approach based on disaggregation to provide forecast probabilities, with hourly resolutions, of electrical consumption, referring particularly to thermal loads for domestic buildings. The study has been validated by using the AMPds dataset. The used Bayesian algorithm is well presented, the metrics are well established, and impressive results are discussed. The perspective of this research is to extend the study for commercial buildings. This is not the first case of adopting probability-based models to approach the NILM problem. In the contribution by Liu et al. [5], an approach based on the probabilistic models to generate an NILM framework is presented. Results allow assessing the high-level flexibility and scalability thanks to the combined classifier modelled as a two-stage decision-making process.

Starting from a clear definition of the NILM problem, in contribution [6], Sykiotis and co-authors discuss the so-called ELECTRIcity approach for the energy disaggregation problem. The strategy is mainly based on transformers and on a pre-training routine and a training routine. The first pre-training step is based on an unsupervised procedure that works with the entire set of data, while the training process routine works for finetuning by using a supervised procedure. The problem is addressed for domestic appliances. The efficiency of ELECTRIcity is remarked by numerous experiments. The attention has been devoted to domestic demands in term of power of kettle, fridges, washers, microwaves, and dishwashers. The dataset UK.DALE-REDD is used. The comparison with other approaches is presented in the paper and the performance evaluation of ELECTRIcity for various sampling time will be an object for future research.

In the contribution [7], Nalmpantis et al. presents an NILM algorithm based on frequency domain techniques; in particular, the use of Fourier Transforms is adopted. The approach, called Neural Fourier Energy Disaggregation (NFED), exploits the paradigm of artificial neural networks. The idea of coupling the power of neural networks with Fourier frequency transform is, hence, emphasized in this paper. Therefore, the disaggregation energy procedure is well tested in the paper and well compared with other methods. The small size of the network and the efficiency of the methods appear promising. A similar approach is based on the analysis of the eigenvalue’s spectrum of the Laplacian representing the network of appliances contributing to energy aggregation. It has been successfully proposed by Ghaffar et al. In [8], it is shown that the robust eigenvalue evaluation algorithm allows the indirect determination of the weight of each load in the measured energy.

The other two contributions are worth mentioning. An advanced approach for realtime NILM and energy disaggregation based on weighted K-nearest neighbors is discussed by Hu et al. in [9]. An interesting problem of reverse engineering has been studied in contribution [10] by Kerk et al., where the NILM method is applied to estimate human activities within a residence using a motif-detection-based approach. Finally, the precious contribution reported in [11] that surveys more of the actual concepts related to NILM and, until now, has been a reference paper for studies in this topic.

In Table 1, some summarizing and correlating aspects among the paper are reported. Each contribution provides new and appealing results. We hope that NILM systems at various level will be integrated in a unique platform in view of conceiving green, sustainable, smart buildings and energy communities.

Indeed, in the last years, the problem of NILM system has been approached by using methods and algorithms covering several advanced systems and control theory results of the last 30 years. This shows how the research in this area covers a wide spectrum from an algorithmic point of view. Moreover, the results obtained are not dependent on the algorithms adopted but rather on how they are applied and adapted on specific NILM problems.

## Figures and Tables

**Table 1:** Summary of the datasets used, problem scales, and the type of approach used in each paper.

	Dataset Used	Problem Scale	Machine Learning	Algorithmic Solution
[1]	AMPds [12], UK-DALE [13], REDD [14], Refi [15]	Small	X	
[2]	COMBED [16]	Large	X	
[3]	UK-DALE [13], REDD [14]	Medium	X	
[4]	AMPds [12]	Medium		X
[5]	REDD [14]	Small		X
[6]	UK-DALE [13], REDD [14], Refi [15]	Small		X
[7]	UK-DALE [13], REDD [14], Refi [15]	Small	X	
[8]	Refi [15]	Small		X
[9]	UK-DALE [13]	Small	X	
[10]	GeLaP [17]	Small		X

## Data Availability

Not applicable.

## References

[1] Kaselimi M., Protopapadakis E., Voulodimos A., Doulamis N., Doulamis A. (2022). Towards Trustworthy Energy Disaggregation: A Review of Challenges, Methods, and Perspectives for Non-Intrusive Load Monitoring. Sensors.

[2] Zhou M., Shao S., Wang X., Zhu Z., Hu F. (2022). Deep Learning-Based Non-Intrusive Commercial Load Monitoring. Sensors.

[3] Hur C.H., Lee H.E., Kim Y.J., Kang S.G. (2022). Semi-Supervised Domain Adaptation for Multi-Label Classification on Nonintrusive Load Monitoring. Sensors.

[4] Massidda L., Marrocu M. (2022). A Bayesian Approach to Unsupervised, Non-Intrusive Load Disaggregation. Sensors.

[5] Liu Y., Wang Y., Hong Y., Shi Q., Gao S., Huang X. (2021). Toward Robust Non-Intrusive Load Monitoring via Probability Model Framed Ensemble Method. Sensors.

[6] Sykiotis S., Kaselimi M., Doulamis A., Doulamis N. (2022). ELECTRIcity: An Efficient Transformer for Non-Intrusive Load Monitoring. Sensors.

[7] Nalmpantis C., Virtsionis Gkalinikis N., Vrakas D. (2022). Neural Fourier energy disaggregation. Sensors.

[8] Ghaffar M., Sheikh S.R., Naseer N., Din Z.M.U., Rehman H.Z.U., Naved M. (2021). Non-Intrusive Load Monitoring of Buildings Using Spectral Clustering. Sensors.

[9] Hu M., Tao S., Fan H., Li X., Sun Y., Sun J. (2021). Non-intrusive load monitoring for residential appliances with ultra-sparse sample and real-time computation. Sensors.

[10] Kerk S.G., Hassan N.U., Yuen C. (2020). Smart Distribution Boards (Smart DB), Non-Intrusive Load Monitoring (NILM) for Load Device Appliance Signature Identification and Smart Sockets for Grid Demand Management. Sensors.

[11] Zoha A., Gluhak A., Imran M.A., Rajasegarar S. (2012). Non-intrusive load monitoring approaches for disaggregated energy sensing: A survey. Sensors.

[12] Bonfigli R., Felicetti A., Principi E., Fagiani M., Squartini S., Piazza F. (2018). Denoising autoencoders for non-intrusive load monitoring: Improvements and comparative evaluation. Energy Build..

[13] Kelly J., Knottenbelt W. (2015). The UK-DALE dataset, domestic appliance-level electricity demand and whole-house demand from five UK homes. Sci. Data.

[14] Kolter J.Z., Johnson M.J. (2011). REDD: A public data set for energy disaggregation research. Workshop on Data Mining Applications in Sustainability (SIGKDD).

[15] Murray D., Stankovic L., Stankovic V. (2017). An electrical load measurements dataset of United Kingdom households from a two-year longitudinal study. Sci. Data.

[16] Batra N., Parson O., Berges M., Singh A., Rogers A. (2014). A comparison of non-intrusive load monitoring methods for commercial and residential buildings. arXiv.

[17] Wilhelm S., Jakob D., Kasbauer J., Ahrens D. (2022). GeLaP: German Labeled Dataset for Power Consumption. Proceedings of Sixth International Congress on Information and Communication Technology.

